# Effect of Extracts Derived from Brown Algae (*Sargassum horneri*) on the Gel Property and Moisture Distribution of Hairtail Surimi Gel (*Trichiurus haumela*)

**DOI:** 10.3390/foods11030411

**Published:** 2022-01-30

**Authors:** Qiuyu Han, Yuxin Wang, Qianqian Chu, Bin Bao

**Affiliations:** 1 College of Food Science and Technology, Shanghai Ocean University, Shanghai 201306, China; fancy_hqy@163.com (Q.H.); terrywyx@icloud.com (Y.W.); achu@kan-pak.com.cn (Q.C.); 2 Shanghai Engineering Research Center of Aquatic-Product Processing & Preservation, Shanghai 201306, China

**Keywords:** brown algae, surimi, gel property

## Abstract

The cross-linking degree between myosin affected the surimi gel properties in the hairtail. In this study, the effects of phlorotannin extracts (PE) derived from brown algae (*Sargassum horneri*) with different concentrations (0.05%, 0.3%, 1% *w/w*) on the hairtail surimi gel-forming properties were investigated in comparison with the commercial phloroglucinol (PG). The breaking forces of surimi gel with 1% PE and 0.05% PG were increased by 14.80% and 2.73%, respectively. The increase in deformation was 9.66% with 1% PE compared with the control added with water, but there was no increase in deformation of surimi gel with 0.05% PG. The improved surimi gel structure with PE as a bridge for the three-dimensional network forming of protein was observed in the microstructure. Moreover, PE could significantly shorten the water relaxation time (*p* < 0.05), reduce free water content (*p* < 0.05), and increase the hydrogen proton density of the hairtail surimi according to the results of NMR, dielectric properties, and MRI map, respectively. Our findings suggest that the extracts from the brown algae could be a potential economical gel structure enhancer to improve the myosin network.

## 1. Introduction

Surimi, a Japanese word for fish mince, has been accepted as a popular raw material for aquatic food. Gel forming was mainly due to the irreversible denaturation of protein resulting in the three-dimensional gel network, in which myosin was essential [1]. Surimi gel was successively produced by the steps of myosin dissolution and crosslink formation. Generally, due to limited resources and economic cost, the fish species with dark flesh or lean fish were increasingly used in producing surimi products, increasing the chance for undesirable fish species to become a popular dish. However, some fish species are unsuitable for the surimi product due to the lack of gelation ability. Species such as hairtail (*Trichiurus haumela*), with high fishing catches in China, was an easy-to-fish and low-cost species, but the low strength of the hairtail surimi gel had a negative impact on product development. As traditional processing of surimi cannot fully promote the myosin unfolding [2], the use of additives to enhance gel properties has been a hot topic in recent years. Some researchers found that gel property intensifiers such as yeast β-glucan [3], oleogels [4], and microbial transglutaminase [5] could prevent surimi gel weakening. Nonetheless, these additives may cause undesirable sensory properties of surimi. Therefore, the search for green and efficient natural food additives to improve the gelation properties of surimi is a current research direction.

Polyphenols, mainly hydroxylated cinnamic acids (e.g., caffeic acid, chlorogenic acid) and flavonols, are compounds found in plant foods that have potential health-promoting effects [6]. Generally, polyphenols are oxidized into quinone during the heating progress and a dimer of which would be formed in the meantime, resulting in a cross-link with an amino acid side chain by C-S or C-N bond formation [7], which affects the gel property during surimi production. Several attempts have been made to study the effects of plant polyphenols on surimi gel properties [8,9]. The effect of phenolic compounds in fast-growing algae on the gel properties of surimi gel has attracted considerable attention. The phenolic compound in brown algae is mainly phlorotannins, a polymer of phloroglucinol [10]. For example, phlorotannins were exclusively possessed in brown algae, with content more than 20% of the dry weight [11]. Jiang et al. illustrated that the phlorotannins from sporophyll extracts worked on myosin under UVA irradiation to increase the crosslinking [12]. Other studies reported that phenolic compounds of seaweed showed no negative affection for sensory properties while increasing the gel property [13,14].

However, these studies only preliminarily explored the effects of algal phenolic compounds on the basic properties of surimi gel, and there is a lack of studies on the gel properties and water retention. Therefore, the enhancement effect of phlorotannins on the surimi gel is worthy of further exploration. In this context, this research aimed to investigate the potential effects of phlorotannins extracted from brown algae (*Sargassum horneri*) on the gel properties and moisture distributions of treated hairtail surimi gel (*Trichiurus haumela*).

## 2. Materials and Methods

### 2.1. Materials

The brown algae (*Sargassum horneri*) were provided by the College of Fisheries and Life Science, Shanghai Ocean University. The hairtail surimi (*Trichiurus haumela*) was purchased from Shanghai Yiyang Yihua Aquatic Products Co., Ltd, Yiyang, China. Phloroglucinol (PG) was of spectral purity grade and the chemicals noted below were of analytical purity grade, purchased from Sinopharm Chemical Reagent Co., Ltd, Shanghai, China.

### 2.2. Preparation of Phlorotannins Extracts

Phlorotannins extracts (PE) was prepared according to the method described in Shitole [13]. Fresh brown seaweed (*Sargassum horneri*) was washed with tap water, followed by draining with gauze, and then dried by an electric thermostatic drying oven at 55 °C until the moisture content was at 2%. The dried seaweed was ground into powder and then sieved using a screen with a diameter of 0.125 mm. Brown seaweed extracts were obtained according to the ultrasound-assisted method of Wang et al. [15]. The powder (1 g) was mixed with 55% ethanol at the ratio of 42:1 (*v*/*w*) and then extracted by ultrasonic extraction for 58 min. The precipitate was collected to repeat the process two more times. The supernatant (100 mL) was fractionated with ethyl acetate (1:1, *v*/*v*) seven times, and the fractions were collected and desiccated with rotary evaporation (Numeric Control Ultrasonic Cleaning Machine, SB4200DT, NingBo XinZhi Biology Science Co., Ltd., Ningbo, China). The pellet was dissolved into ethanol with different concentrations for further use.

### 2.3. Phlorotannins Extracts Identification

PE was identified by liquid chromatography-time of flight-mass spectrometry (LC-TOF-MS) (1290LC-6224, Agilent Technologies, Inc., Beijing, China) according to the method in Ferreres [16] with some modifications.

#### 2.3.1. Chromatographic Conditions

The column used was a YMC-Pack ODS-AQ C18 (75 × 4.6 mm, 3 μm). The column temperature was set at 20 °C. Mobile phase: 0.1% formic acid methanol was used as phase A and 0. 1% formic acid acetonitrile as phase B. The elution program was set to gradient elution: 0–5 min: 0% B, 15 min: 30% B, 17.5–20 min: 80% B, 22.5–26 min: 0% B. The flow rate was 0.3 mL/min, the injection volume was 5 μL, and the injection temperature was 4 °C. The DAD wavelength scanning range was 200–400 nm, and the UV detection wavelength was 280 nm.

#### 2.3.2. Mass Spectrometry Conditions

The mass spectrometry was performed at a flow rate of 8.0 L/min, a drying gas temperature of 350 °C, an atomization gas pressure of 40 psig, a lysis voltage of 175 V, and a capillary voltage of 4000 V. The mass spectra were scanned in the range of *m*/*z* 100~1800.

Mass calibration: The reference solution was introduced into the ion source simultaneously with the sample through the reference Nebulizer for real-time reference. The ions 322.048132, 622.028960, and 922.009798 were selected in positive ion mode.

### 2.4. Preparation of Surimi Gel

The purchased surimi was cut into small pieces (5 cm × 5 cm) after thawing in a 4 °C refrigerator until the core temperature reached 0–2 °C. It was then chopped under 10 °C for 15 min. The final moisture content of surimi was adjusted to 78.30% by adding cold distilled water. Then PE and PG (5.00 mL) at various concentrations (0.05%, 0.3%, and 1%) dissolved into 5% ethanol were added, and the distilled water was used as the blank control. Next, the homogeneous surimi sol was obtained after chopping for 3 min under 4 °C. To prepare the surimi gel, the sol with PE and PG were stuffed into a 20 mm diameter casing and both sides were sealed tightly. It was then heated at 35 °C for 3 h and subsequently incubated at 90 °C for 30 min in a water bath. All heated surimi gel was rapidly cooled in ice water and stored at 4 °C for analysis. PG was used as a positive control.

### 2.5. Texture Analysis of Surimi Gel

The gel strength of surimi gel was carried out using a texture analyzer (TA.XT Plus, Stable Micro System, Surrey, UK) as described by Julavittayanukul [17] with slight modifications. The surimi gel was cut into a cylinder (20 × 30 mm) after equilibrating at room temperature before analysis. The aspherical plunger (P/5S) was pressed into the surimi gel surface perpendicularly with a constant force (10 g) and a stable rate (1 mm/s) at a certain distance from the surface (20 mm). The quality of the gels was assessed by measuring their breaking force (g) and deformation (cm). Gel strength was calculated according to the equation:

Gel strength = breaking force (g) × deformation (cm)

### 2.6. Scanning Electron Microscopy (SEM) of Surimi Gel

The microstructure of prepared surimi gel was measured by SEM according to the method in Oujifard [18] with minor modifications. Briefly, the samples (3 mm × 3 mm × 2 mm) were fixed with glutaraldehyde at 2.5% (*v*/*v*) and incubated for 24 h at room temperature, followed by filtration. The precipitates were rinsed with 0.2 M phosphate buffer (pH 7.2) three times. The samples were gradient eluted with ethanol at different concentrations of 50%, 60%, 70%, 80%, and 90% for 20 min, and then washed with 100% ethanol for 30 min (three times). The dehydrated samples were mounted on a conduct electric stub in the sample treatment stage after vacuum freeze drying, then sputter-coated with gold. The microstructure of prepared surimi gel was available for observing and recording under scanning electron microscopy.

### 2.7. Dielectric Properties Determination of Surimi Gel

The dielectric constant and dielectric loss factor of each surimi gel sample were determined using the coaxial probe coupled with a PNA-L Network Analyzer (N5230C, Agilent Technologies Inc., Santa Clara, CA, USA), according to the method in Zhang et al. [19], with minor modifications. To maintain stability and minimize the error, the measurement system was kept in a stable environment for 2 h before calibration and measurement, followed by adjustment under three different types of loads: open-air, short circuit, and 25 °C deionized water. In the present study, a frequency range of 300–4500 MHz was used to determine the dielectric properties of control and treated samples under 20 °C. Each replicate was determined three times to obtain the mean value.

### 2.8. Nuclear Magnetic Resonance (NMR) Analysis of Surimi Gel

The water state of the treated surimi gel samples could be observed by determining spin-spin water relaxation time (T2) with carr-purcell-meiboom-gill (CPMG) pulse sequence using low-field NMR relaxation measurement (MesoMR 23-060H.I, Niumag Electric Corporation, Shanghai, China). Surimi gel was prepared in a cylinder with a diameter of 20 mm and a height of 30 mm at room temperature, and the gel with PG was prepared in a square container with a length of 20 mm and a height of 30 mm. The spin-spin relaxation time (T2) was measured via the CPMG pulse sequence. Parameter settings: SFI = 2 MHz, P1 = 17 μs, SW = 100.00 kHz, RFD = 0.08 ms, NS = 16, P2 = 33 μs, TW = 2500 ms, TE = 0.60 ms, and NECH = 3000.

### 2.9. Magnetic Resonance Imaging (MRI) Analysis of Surimi Gel

Proton density-weighted images were acquired on a MesoMR23-060H-I NMR coopered with an imaging system (Niumag Electric Corporation, Shanghai, China). The test samples were placed in the center of the radio frequency coil at the center of the permanent magnetic field, and the hydrogen proton density image of the surimi gel was obtained by multiple spin echo (MSE) imaging sequences. According to the previous measurement of T2 relaxation time with the CPMG sequence, the repetition time was 500 ms, the spin-echo time was 20 ms, and the center frequency was 21 MHz.

### 2.10. Statistical Analysis

SPSS 22 and Origin 2017 were used to analyze experimental data. All experiments were performed three times with the hairtail surimi gel of the same batch. All obtained data were reported in the form of mean ± SD and differences were considered significant at *p* < 0.05. One-way analysis of variance (ANOVA) was used and mean comparison was carried out using Tukey’s multiple range test. The optimization of PE extraction was based on the Box–Behnken (BBD) method.

## 3. Results and Discussion

### 3.1. PE Identification

The brown algae extract was identified using LC-TOF-MS, and the composition is shown in Table 1. 

From Table 1, the average molecular weights of Nos. 78 and 96 are 250.2258 and 374.3036, respectively, with theoretical values of 250.2042 and 374.2984 and molecular formulae of C_12_H_10_O_6_ and C_18_H_14_O_9_, respectively, which are likely to be dimers and trimers of phloroglucinol (PG).

The molecular ion peak of compound No. 96 in positive ion mode with a retention time of 14.8 min was *m*/*z* 375 [M+H]^+^ with an average molecular weight of 374.3036; the theoretical value was 374.2984 and the molecular formula was C_18_H_14_O_9_. The molecular ion was cleaved by electrospray ionization to fragment peaks with *m*/*z* 357 and 232, where the removal of water resulted in the characteristic fragment *m*/*z* 357 [M+H-H_2_O]^+^, and the removal of phloroglucinol and hydroxyl oxygen resulted in the ionic fragment *m*/*z* 232 [M+H-O-C_6_H_6_O_3_]^+^. The results indicated that this compound was a trimer of phloroglucinol linked by C-O-C oxidized phenols.

The extraction rate and purity of brown algae extract are shown in Table 2. 

The extraction rate of PE was 25.16% with the optimized ultrasound-assisted extraction method, which showed a significant increase over microwave extraction (19.96%) and Soxhlet extraction (13.13%) [15]. The phlorotannin were only 6.46% of the dry matter in this study, which was lower than that of green algae. However, the high purity of PE (73.85%) indicated that the major components of PE was phlorotannin, which mainly contributed to the enhanced protein cross-linking in surimi gel.

### 3.2. Gel Property of Surimi Gel

PE and PG were dissolved in 5% ethanol and the effects of the added solvent on surimi gel were investigated. Our results found that the gel strength of surimi gel with 5% ethanol (3766.60 ± 261.60 g·cm) had no significant difference compared with the control sample (3623.80 ± 86.10 g·cm) (*p* > 0.05), suggesting that the 5% ethanol had little effect on surimi gel strength.

**Figure 1:**
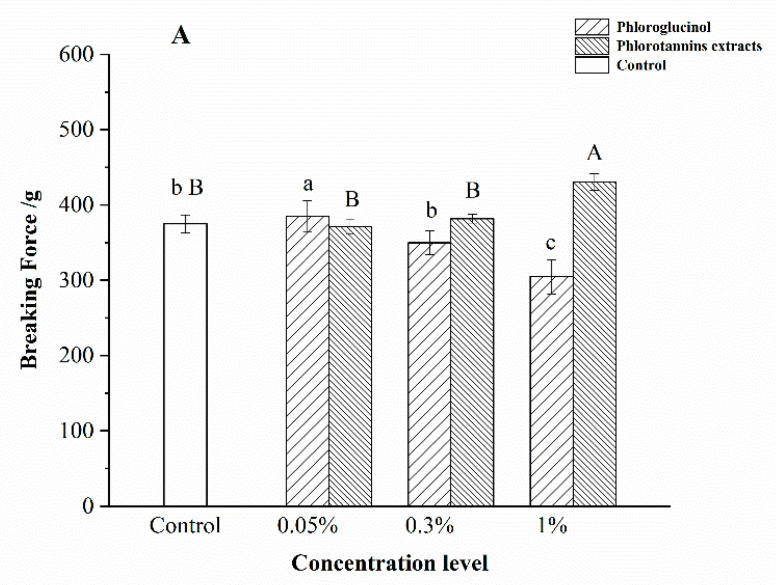

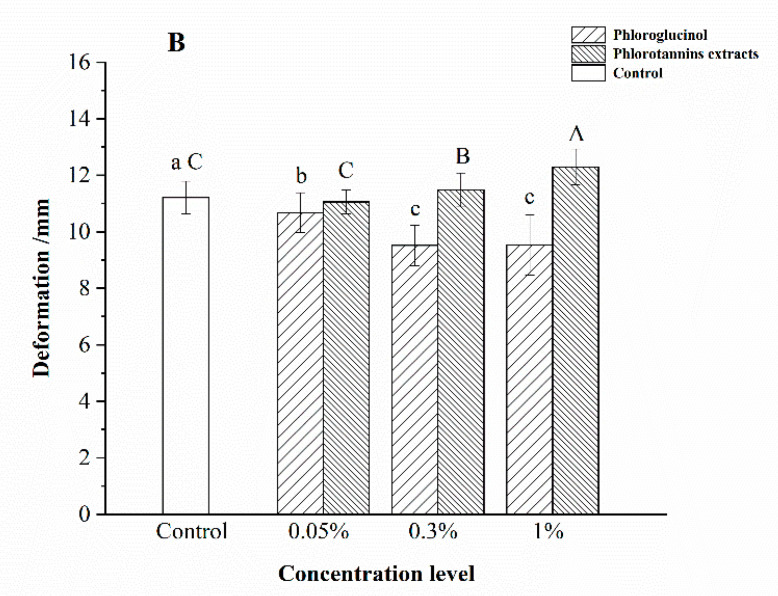
Effect of phlorotannins extracts (PE) and phloroglucinol (PG) on the breaking force (**A**) and deformation (**B**) of surimi gel. Bars represent the standard deviation (*n* = 3). Uppercase letters indicated the significant difference (*p* < 0.05) between different concentrations of PE. Lowercase letters indicated the significant difference (*p* < 0.05) between different concentrations of PG.

The effects of different concentrations of PG and PE on the gel properties of hairtail surimi gel are shown in Figure 1. 

The breaking force of surimi gel treated by PE showed a concentration dependence (Figure 1A). Compared with the control, the breaking force of surimi gel treated with 1% PE was improved significantly (*p* < 0.05). The highest breaking force of PG-treated samples was observed in the 0.05% PG-treated one, significantly higher than that of the control (*p* < 0.05). However, 1% PG decreased the breaking force significantly (*p* < 0.05), with an 18.73% decrease (Figure 1B). In addition, the deformation of surimi gel increased with the increase of PE concentration. Compared with the control group, the deformation of samples with 1% PE significantly increased (*p* < 0.05), whereas the deformation of samples with 1% PG decreased by 12.94%. Theoretically, the gel was formed due to the phenolic compounds forming a bridge between the myosin cross-linking [12]. In contrast, the phenolic compounds were oxidized into quinones under high temperature [20], followed by inducing the protein unfolding to provide more hydrophobic binding sites for the quinones [21]. Moreover, quinone was an electrophile intermediate that underwent a secondary addition reaction once attacked by a nucleophilic molecule [20], leading to the formation of cross-linked protein polymers. Therefore, 1% PE and 0.05% PG could form a rigid molecular structure with the unfolded myosin, which enhanced the surimi gel property.

Notably, the increase in the dosage of PE and PG showed opposite effects on the gel properties, which suggested that PG and PE might interact with the myosin of surimi gel in different ways. There are two distinguished complexation mechanisms for additives and myosin: high-dosage dependence monodentate and low-dosage dependence multidentate [22,23]. According to the results in Figure 1, it is suggested that PE was monodentate. Phenolic compounds with a monodentate complexation mechanism showed significant enhancement of the cross-linking between proteins and phenolic compounds with high concentrations [22,23]. In contrast, the multidentate mechanism required multiple binding sites in phenolic compounds. In the present study, higher deformation and breaking forces of surimi gel were observed at a lower level of PG (0.05%), indicating that PG was a multidentate mechanism on myosin. At the same time, the self-aggregation of phenolic compounds with increasing concentration was formed [20], and the hydroxyl group of bonding amino acid residues was decreased, which negatively affected the surimi gel properties. Balange et al. reported that the increased dosage of oxidized phenolics decreased the gel strength of mackerel surimi gel, supporting the results of the present study [24].

### 3.3. The Microstructure of Surimi Gels with Phlorotannins Extraction and Phloroglucinol

**Figure 2:**
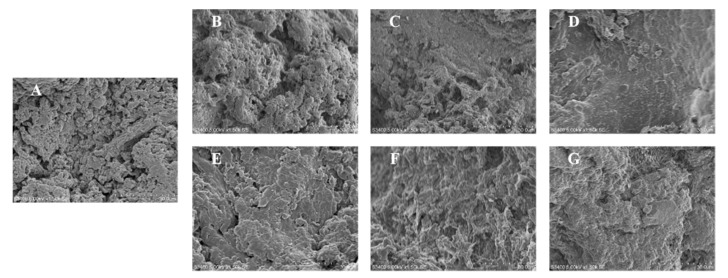
Scanning electron microscopy results of hairtail surimi gel with different concentrations of phlorotannins extracts (PE) and phloroglucinol (PG). (**A**) control sample; (**B**) surimi gel with 0.05% PG; (**C**) surimi gel with 0.3% PG; (**D**) surimi gel with 1% PG; (**E**) surimi gel with 0.05% PE; (**F**) surimi gel with 0.3% PE; (**G**) surimi gel with 1% PE.

The microstructure is important in the surimi gel network, which can be observed by SEM [25]. The SEM can reflect the relationship and the combination degree of protein-protein and protein-water [26]. The microstructures of hairtail surimi gel of different concentrations of phlorotannins extracts and phloroglucinol are illustrated in Figure 2.

Surimi gel samples with PE (Figure 2E–G) had different results from samples with PG. Surimi gel surface became coarser with increasing concentrations of PE. As shown in Figure 2G, the cross-linking of the protein fiber was the most compact with 1% PE, compared with the control sample. The results are similar to the report of Balange [27] that the tannins could develop the surimi gel with increasing dosage. Figure 2B shows that the cross-linking formed between myosin and 0.05% PG. Maqsood [28] suggested that the forming of cross-linking between PG and surimi gel was attributed to the covalent binding. However, Figure 2C illustrates that the bigger surface cavities appeared with the 0.3% PG, and the protein fiber almost disappeared in the 1% PG treated sample (Figure 2D). It was inferred that the PG aggregation was the major factor to inhibit the protein linkage and the protein-phenolic covalent-binding forming [29]. The results of Figure 2 also reveal that PE and PG had different connected approaches to the surimi gel structure. PE could induce the surimi gel surface compactness with the increasing dosage, and its effect was better than the PG treated sample in optimal concentration. The developed structure was attributed to a great number of binding sites provided by PE under the monodentate reaction mechanism, which was consistent with the results of gel properties of hairtail surimi gel shown in Figure 1.

### 3.4. Dielectric Properties of Surimi Gel

The dielectric property of food is defined as the interaction between the medium and electromagnetic field and is expressed in terms of complex relative dielectric permittivity (ε*) [30]. The value of ε* was used to represent the medium dielectric property for the real part and imaginary part called dielectric constant (ε′) and dielectric loss factor (ε″), respectively [31], which existed in the time-change high-frequency sinusoidal field or microwave field. In addition, the ε″ was characterized by different relaxation phenomena [32], which were relative to the dipolar property of the water molecule. The dielectric property of water was treated as the main cause of dielectric loss and dielectric absorption. Mabrook [33] reported that the conductance of full-fat milk increased with the increase in water. The ε″ was described by Equation (1) (Debye relation), reflecting different relaxation phenomena with different effects in food with high moisture content.

Ε″ = εd″ + σ/ε0ω(1)

From Equation (1), ε″ represented the loss factor, εd″ represented the relaxation phenomenon caused by dipolar polarization, and σ/ε0ω represented the relaxation phenomenon caused by ionic conductivity. Normally, the moisture content of surimi gel was over 70%. Thus, the dielectric behavior could be reflected by the Debye relation for the properties of free water in surimi gel.

**Figure 3:**
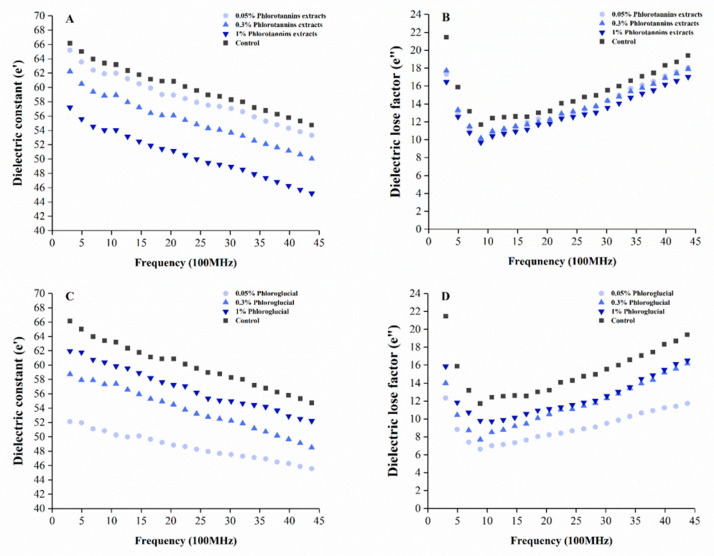
The effect on dielectric constant and dielectric loss factor of phlorotannins extracts (**A**,**B**) and phloroglucinol (**C**,**D**) to hairtail surimi gel.

The effects on ε′ and ε″ of hairtail surimi gel with PE and PG at different levels are shown in Figure 3. 

Compared with the control, both ε′ and ε″ were decreased by PE and PG. The ε′ decreased with both the PE and the frequency increasing (Figure 3A), whereas Figure 3C shows that a lower concentration of PG promoted ε′ decreasing of surimi gel. As shown in Figure 3B, ε″ markedly decreased with increasing frequency and then ε″ reached a minimum at 900 MHz, followed by a slower increase in 1000–4500 MHz, which was the microwave frequency domain. The lowest ε″ was observed in surimi gel with 1% PE among the three concentrations. From Figure 3D, the minimum ε″ value of surimi gel with PG also appeared at 900 MHz. The changing tendency of ε″ with different concentrations was similar. The ε″ value of 0.05% PG treated surimi gel was the lowest among all the samples.

The ε′ depends on the frequency of microwave radiation and decreases with the increase in frequency. Water is a polar molecule, and it will be oriented or polarized in an external electric field [34,35]. The denser gel structure of surimi gel formed more hydrogen bonds, thus limiting the mobility of water molecules. Therefore, the results of ε′ shown in Figure 3A,C indicated that a high concentration level of PE (1%) promoted the increase in bound water in the surimi gel, resulting in the diminishing of the dielectric constant. However, the degree of restriction to the water of PG showed a negative dose-dependent manner.

**Figure 4:**
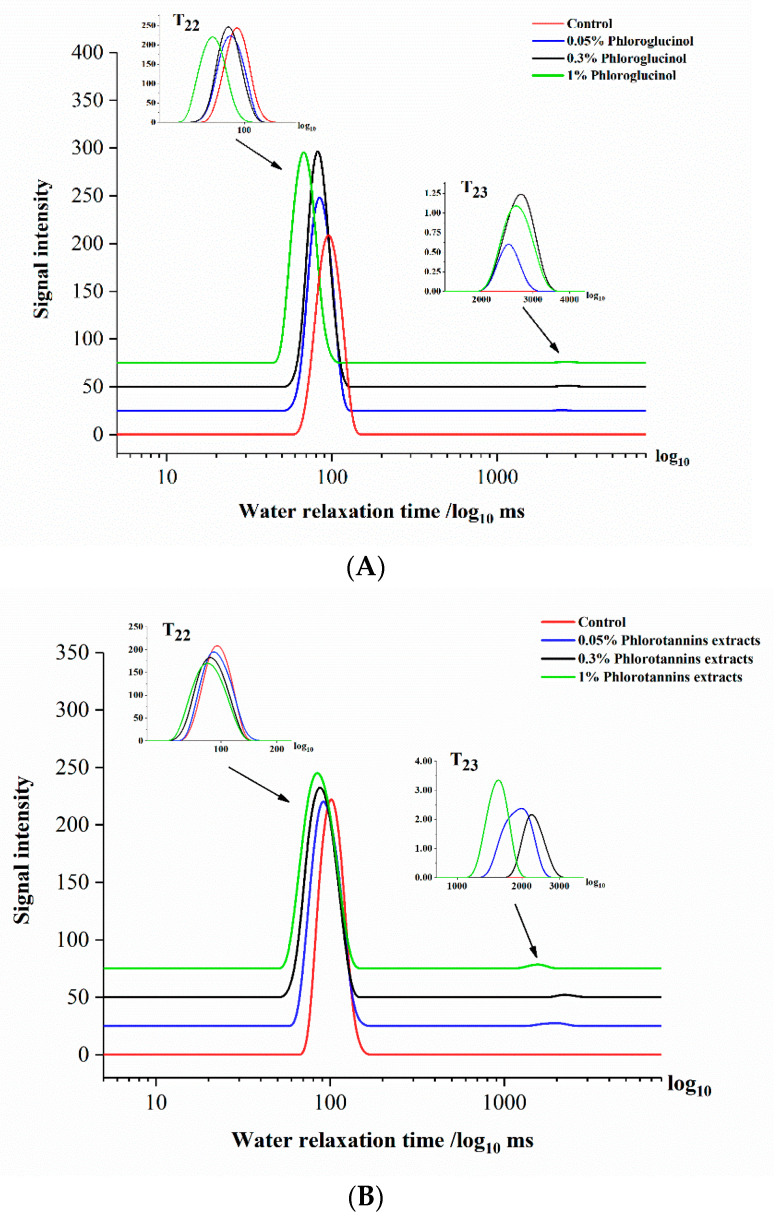
The water relaxation time of hairtail surimi gel with different concentrations of phloroglucinol (PG) (**A**) and phlorotannins extracts (PE) (**B**).

When it came to then ε″, the results are shown in Figure 3B, and Figure 3D indicated that the main influenced factor of dielectric loss in lower frequency differed from that in the frequency higher than 1000 MHz. The common effect of εd″ and σ/ε0ω was identified as the main reason for the increase of ε″ in a lower frequency, which promoted the ε″ decreasing rapidly, as shown in Figure 3B,D. In contrast, at a frequency above 1000 MHz, the εd″ contributed the most to the ε″ increase among the four types of polarization [36]. Once the frequency was higher than the inflection point (900 MHz in this study), the ε″ change was well described by the Debye equation. That is, with the increase in frequency shown in Figure 3B and Figure 4B, the polarized dipole was stopped from rotating up with the change of electric field [37] and then resulted in the gradual increase in the ε″ value. The εd″ in surimi gel detected in the high-frequency domain was mainly due to the change of free water. Therefore, the restriction of free water was promoted by the additives in surimi gel. Clarion [38] illustrated that higher water activity in food was derived from the higher dielectric property. A higher ε″ indicated a higher free water content in surimi gel, reflecting the higher water activity of surimi gel treated by the lower concentration level of PE (0.05%) and higher PG (0.3% and 1%). The difference in ε″ value emphasized the different protein-binding abilities between PE and PG. Stronger covalent interaction between 1% PE/0.05% PG and protein in surimi gel was deducted due to the stronger restriction of water, which provided support to the change of breaking force and deformation shown in Figure 1 and the surface change in Figure 2. However, the effects of electric properties on moisture state were unclear, and further analysis is necessary.

### 3.5. The Water Relaxation Time of Surimi Gel

For surimi gel property research, an intuitive and fast testing instrument was needed. Low field nuclear magnetic resonance (LF-NMR) is non-destructive, allows for rapid detection, has a low sample requirement, and can be used to determine multiple properties in food such as moisture, protein, and texture [39]. It was well-known that the water-binding state plays a vital role in gel-forming, and NMR has been applied to non-destructively explore the moisture mobility and diffusion of different water types in high water content samples [40,41]. Several studies showed that water-holding capacity and water-binding ability were mainly due to the change in myofibrillar protein during the heating progress [42,43]. When proteins are surrounded by water molecules in a specific binding location, transversal relaxation time (T2) of NMR can be considerably influenced if these water molecules exchange rapidly enough in solution. The exchange can be regarded as a diffusion process. The length of relaxation time is related to the existence of hydrogen protons and the physical and chemical environment [44]. Therefore, water 1H T2 was used to reflect the protein mobility and the interaction between protein and water. Empirically, three independent peaks were fitted exponentially by NMR spin-spin relaxation signal. In the present study, two separate signal peaks were observed in all samples by CPMG sequence: bond water (T22) and free water (T23). The transverse relaxation time of different treated surimi gels are shown in Figure 4.

As Figure 4B shows, the T22 relaxation time of the 1% PE sample shifted sharply left to a shorter response time. The samples treated by 0.3% and 0.05% PE showed no significant difference in response time compared with the control (*p* > 0.05). Moreover, it was observed that the T23 peak of 0.05%, 0.3%, and 1% PE was also shifted to the left. Compared with the control, similarly significant shifts in T22 and T23 were observed in PG-treated samples (Figure 4A), which were consistent with the analysis of ε′ shown in Figure 3A,C. The results indicated that the water movability was reduced in the surimi gel network by adding PE (1%) and PG (0.05%). The peak position of relaxation time shift to the left was attributed to the greater hydrogen proton binding force or the smaller degree of water freedom [45]. The water in the outer layer could be quickly reoriented with a high degree of freedom, compared to the inner layer of water with a stable and tight binding state. Hence, the internal three-dimensional network structure of surimi gel tended to be more stable with PE and PG added.

It is worth noting that the T23 relaxation time shift of surimi gel with added PG and PE was different. The NMR T23 relaxation times of surimi gel with added PE (0.05%, 0.3%, and 1%) were 2.56 s, 2.71 s, and 2.92 s, respectively, and those of PG (0.05%, 0.3%, 1%) treated surimi gel were 0.90 s, 1.49 s, and 1.20 s, respectively. The 1% PE and 0.05% PG induced T23 relaxation time of surimi gel shifted more to the left than other concentrations, indicating that the corresponding free water was reduced. The proportion of free water is inversely proportional to the strength of the gel structure [46]. Therefore, the gel property of the surimi gel sample was enhanced by the addition of 1% PE and 0.05% PG, which supported and supplemented the change of ε′ and ε″ of surimi gel with added PE and PG, shown in Figure 3. Regarding the difference between PE and PG, the main factor was the different ways that two kinds of phenolic compounds interacted with myosin, resulting in various degrees of tightness in the network of the treated surimi gel, which supported the results shown in Figure 1.

### 3.6. Water Distribution of Surimi Gel

**Figure 5:**
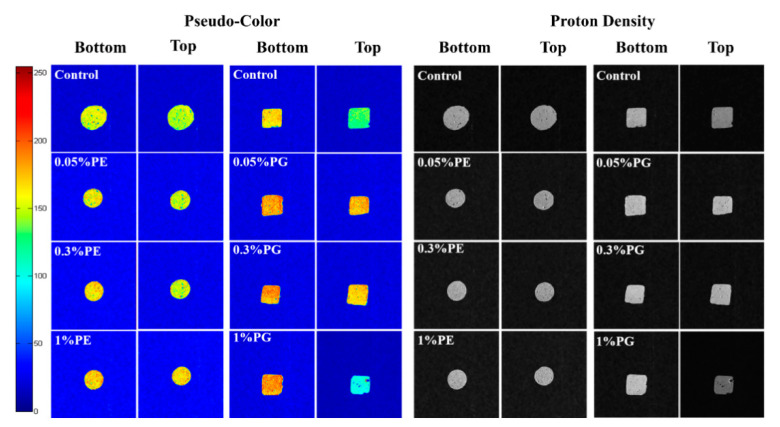
Water distribution of hairtail surimi gel with different concentrations of phlorotannins extracts (PE) and phloroglucinol (PG).

In muscle tissues, the state of moisture was characterized by MRI [47]. MRI is regarded as a non-destructive and precise method, widely used in the food storage and processing fields in recent years. Figure 5 shows the changes in water distribution of the hairtail surimi gel structure treated by different concentrations of PE and PG. 

After weighted imaging, all aqueous phases in the samples were characterized by MRI techniques [48]. Hydrogen proton density indicated the distribution of water. The pseudo-color map obtained after the weighting process clearly shows the hydrogen proton density difference according to the color change, which highlights the different water phases in the gel. The color bar serves as a reference for color changes in pseudo-color: a higher hydrogen proton density represents a redder pseudo-color.

As Figure 5 shows, the surimi gel sample treated by 0.05% PE showed little difference compared with the control sample. However, the sample treated by 0.05% PG increased hydrogen proton density remarkably, which indicated that the three-dimension network of surimi gel was greatly enhanced with a lower concentration of PG. The bottom of the surimi gel treated by 1% PG could be observed with more hydrogen protons. Nevertheless, it was found from the pseudo-color figure that the density of hydrogen protons on the sample surface was much higher than that on the bottom. It could be suggested that the poor water-holding capacity of gel samples treated with 1% PG resulted in different moisture distributions at the top and bottom layers [49]. It was observed from the proton density picture that samples treated with a high dosage of PG were darker at the top than at the bottom. However, the sample treated with PE exhibited good structure uniformity indicating the tight combination of PE and surimi gel protein. These results proved that 1% PE and 0.05% PG contributed more to strengthening the gel property.

## 4. Conclusions

Two different types of polyphenolic compounds showed dramatically different effects on hairtail surimi gel. Because of the different interaction mechanisms with myosin of surimi gel, 1% PE and 0.05% PG exhibited a comparatively strengthening influence on surimi gel, with PE showing a better performance. Therefore, 1% of PE could be treated as an optimized option to enhance the hairtail surimi gel. PE could be treated as a potential economic resource for surimi gel production. However, there was also some inadequacies, necessitating supplementary research in the present study. Myosin was a crucial factor for gel-forming, so the sulfate-polyacrylamide gel electrophoresis could be used to further investigate myosin change in surimi gel with added phlorotannins. According to the results of low-field NMR, optimal concentrations of PG and PE would significantly increase the gel quality of surimi gel. Nevertheless, a more sufficient method to reflect the relationship of examined samples and the property needs to be established.

## Figures and Tables

**Table 1:** The composition of brown algae extracted by LC-TOF-MS.

Number	Retention Time/min	*m*/*z*	Average Molecular Weight	Chemical Formula
1	2.620	182.96170	182.64745	
2	2.628	124.99404	124.38192	
3	2.647	101.00841	100.37207	
4	2.657	110.00857	109.35370	
5	2.690	112.96627	112.07419	
6	2.720	198.93934	198.38203	C_4_H_6_OS_4_
7	2.721	116.98560	116.00102	
8	2.729	128.94901	128.02619	C_4_OS_2_
9	2.734	328.89734	328.38389	C_10_H_4_N_2_OS_5_
10	2.737	458.85542	458.34203	C_13_H_2_N_2_O_7_S_5_
11	2.742	474.83270	474.04785	
12	2.744	214.91687	213.99547	C_9_HC_l3_
13	2.746	344.87480	344.02586	
14	2.746	140.91655	139.98820	
15	3.179	256.04484	233.19057	C_9_H_7_N_5_O_3_
16	3.183	421.11386	420.30871	C_13_H_20_N_6_O_8_S
17	3.187	302.04970	301.19281	C_11_H_11_NO_9_
18	3.190	329.18305	328.33031	C_14_H_24_N_4_O_5_
19	3.195	405.13736	404.63680	C_22_H_28_OS_3_
20	3.199	345.1552	344.48908	C_17_H_28_O_3_S_2_
21	3.2	172.09316	171.15199	C_11_H_24_N_6_O_4_S
22	3.22	337.16384	336.30625	C_8_H_5_N_5_O
23	3.226	188.05795	187.14249	C_14_H_28_N_2_O_4_S
24	3.234	321.18461	320.64899	C_6_H_13_N_3_OS_2_
25	3.271	208.05698	207.04968	C_8_H_6_C_l_N_5_O
26	3.277	224.0342	223.56814	C_14_H_21_C_l_N_6_O_4_
27	3.281	373.13822	372.7625	C_8_H_7_N_5_O_5_S
28	3.352	286.02447	285.20045	C_17_H_26_N_2_O_3_
29	3.361	307.20102	306.34703	C_11_H_24_N_6_O_4_S
30	3.361	270.04693	269.46882	C_9_H_19_NS_4_
31	3.368	208.05658	207.21412	C_5_H_9_N_3_O_6_
32	3.369	158.09690	157.15869	C_11_H_11_N
33	3.433	224.03409	223.49965	C_8_H_6_ClN_5_O
34	3.437	373.13812	372.74777	C_13_H_25_ClN_2_O_8_
35	3.445	172.09212	171.15013	C_4_H_9_N_7_O
36	3.448	337.16171	336.31311	C_13_H_24_N_2_O_8_
37	3.451	188.05770	187.1416	C_8_H_5_N_5_O
38	3.503	321.18443	321.28052	C_16_H_30_C_l2_N_2_
39	3.528	235.07545	234.48632	C_9_H_10_N_6_S
40	3.529	251.05280	250.17086	C_8_H_6_N_6_O_4_
41	3.821	166.10571	165.19649	C_6_H_15_NO_4_
42	3.870	194.13675	193.27074	C_8_H_19_NO_4_
43	3.932	307.20089	306.32947	C_17_H_26_N_2_O_3_
44	4.099	150.17505	149.23387	
45	4.143	337.16147	336.30020	C_14_H_2_0N_6_O_4_
46	4.291	307.20132	306.34264	C_17_H_26_N_2_O_3_
47	4.914	176.12795	175.23936	C_8_H_17_NO_3_
48	4.914	194.13837	193.23043	C_8_H_19_NO_4_
49	5.809	303.18921	302.29168	C_10_H_22_N_8_O_3_
50	8.410	123.05531	122.12213	C_6_H_6_N_2_O
51	9.828	260.14681	237.31662	C_10_H_23_NO_5_
52	10.798	194.13863	193.24705	C_8_H_19_NO_4_
53	10.816	324.24887	323.39376	C_14_H_33_N_3_O_5_
54	10.816	411.31749	410.31017	C_18_H_42_N_4_O_6_
55	10.825	455.34358	454.61986	C_20_H_46_N_4_O_7_
56	10.825	352.24382	351.41140	C_15_H_33_N_3_O_6_
57	10.827	238.16453	237.30869	C_10_H_23_NO_5_
58	10.827	176.12787	175.25413	C_8_H_17_NO_3_
59	10.832	368.27506	367.46745	C_16_H_37_N_3_O_6_
60	10.841	499.37012	498.6614	C_22_H_50_N_4_O_8_
61	11.108	102.1278	101.18727	C_6_H_15_N
62	11.180	304.17291	281.31975	C_12_H_27_NO_6_
63	11.181	113.03499	112.14134	C_4_H_4_N_2_O_2_
64	11.324	116.07051	115.11642	C_5_H_9_NO_2_
65	11.339	200.0885	199.18191	C_5_H_9_N_7_O_2_
66	11.422	314.09103	313.32248	C_11_H_15_N_5_O_4_S
67	11.518	136.06170	135.12716	C_5_H_5_N_5_
68	11.632	323.16347	322.45909	C_13_H_26_N_2_O_5_S
69	11.680	127.05003	109.07874	C_5_H_3_NO_2_
70	12.116	116.07110	115.12176	C_5_H_9_NO_2_
71	12.117	233.14945	232.27569	C_10_H_20_N_2_O_4_
72	12.488	228.06569	227.26876	C_6_H_9_N_7_OS
73	12.496	174.05757	173.21876	C_7_H_11_NO_2_S
74	12.506	172.09389	171.15761	C_4_H_9_N_7_O
75	12.992	217.10404	194.21772	C_8_H_18_O_5_
76	13.006	162.11199	161.22416	C_7_H_15_NO_3_
77	13.008	277.17483	276.31391	C_12_H_24_N_2_O_5_
78	13.135	251.05470	250.22581	C_12_H_10_O_6_
79	13.178	321.20715	320.41285	C_20_H_24_N_4_
80	13.735	321.20217	320.36389	C_14_H_28_N_2_O_6_
81	13.898	133.08577	132.16099	C_6_H_12_O_3_
82	13.900	239.14873	238.28483	C_10_H_22_O_6_
83	13.940	136.06160	135.12835	C_5_H_5_N_5_
84	13.942	298.09683	297.30583	C_11_H_15_N_5_O_3_S
85	14.077	109.02839	108.07940	C_6_H_4_O_2_
86	14.357	197.11603	196.24830	C_11_H_16_O_3_
87	14.358	141.05368	140.13538	C_7_H_8_O_3_
88	14.358	179.10562	178.20740	C_11_H_14_O_2_
89	14.359	237.10761	214.23068	C_7_H_14_N_6_O_2_
90	14.359	133.10010	132.28006	C_10_H_12_
91	14.369	172.09028	171.25490	C_7_H_13_N_3_S
92	14.507	130.15875	129.27435	C_8_H_19_N
93	14.634	283.17414	282.32968	C_12_H_26_O_7_
94	14.637	133.08560	132.21589	C_6_H_12_O_3_
95	14.762	161.10666	160.20811	C_10_H_12_N_2_
96	14.802	375.07051	374.30363	C_18_H_14_O_9_
97	15.243	327.20070	326.37497	C_11_H_30_N_6_OS_2_
98	15.256	205.13307	204.23334	C_12_H_16_N_2_O
99	15.609	205.13317	204.24578	C_12_H_16_N_2_O
100	15.779	388.25120	370.41506	C_12_H_30_N_6_O_7_
101	16.179	236.16439	235.31530	C_14_H_21_NO_2_
102	16.250	432.27712	414.47561	C_14_H_34_N_6_O_8_
103	16.306	330.16396	329.38774	C_18_H_23_N_3_OS
104	16.321	360.23156	359.39133	C_25_H_29_NO
105	16.337	209.14770	208.28387	C_8_H_20_N_2_O_4_
106	16.343	133.09741	132.20609	C_5_H_12_N_2_O_2_
107	16.593	212.11430	211.24952	C_8_H_13_N_5_O_2_
108	16.666	476.30475	458.52634	C_16_H_38_N_6_O_9_
109	16.757	219.14800	218.34457	C_13_H_18_N_2_O
110	16.922	207.09448	206.25775	C_11_H_14_N_2_S
111	17.042	520.33041	502.58621	C_18_H_42_N_6_O_10_
112	17.070	248.14593	247.25591	C_7_H_17_N_7_O_3_
113	17.301	350.23023	349.45141	C_16_H_27_N_7_O_2_
114	17.384	564.35756	546.65633	C_21_H_42_N_10_O_7_
115	17.699	608.38428	607.72716	C_23_H_49_N_11_O_8_
116	17.733	542.25877	541.55646	C_23_H_31_N_11_O_5_
117	17.799	123.11549	122.21653	C_9_H_14_
118	17.801	207.13640	206.28160	C_9_H_14_N_6_
119	17.828	447.09129	446.36504	C_21_H_18_O_11_
120	17.894	270.12425	269.74843	C_14_H_20_ClNO_2_
121	18.366	321.09245	320.28310	C_11_H_16_N_2_O_9_
122	18.378	219.10016	218.36085	C_9_H_18_
123	18.381	524.24689	506.59667	C_30_H_34_O_5_S
124	18.596	588.40820	565.78566	C_29_H_59_NO_9_
125	18.784	388.28160	387.57821	C_20_H_33_N_7_O
126	18.830	135.11497	134.24089	C_10_H_14_
127	18.831	197.11686	196.23207	C_11_H_16_O_3_
128	18.831	133.10030	132.20364	C_10_H_12_
129	18.835	179.10436	178.22762	C_7_H_10_N_6_
130	18.872	384.28912	383.56288	C_25_H_37_NO_2_
131	18.919	365.11881	364.31631	C_13_H_20_N_2_O_10_
132	18.971	406.21052	405.94931	C_18_H_32_ClN_3_O_5_
133	19.134	218.20622	217.33160	C_8_H_23_N_7_
134	19.146	701.49011	700.91185	C_32_H_68_N_4_O_12_
135	19.150	679.50780	678.95367	C_31_H_66_N_8_O_8_
136	19.235	186.21576	185.37423	
137	19.295	508.32521	507.66241	C_25_H_37_N_11_O
138	19.366	538.37259	537.69005	C_27_H_43_N_11_O
139	19.380	197.11712	196.22811	C_11_H_16_O_3_
140	19.544	559.37285	558.74173	C_29_H_42_N_12_
141	19.560	242.28420	241.44480	C_16_H_35_N
142	19.670	274.27228	273.44389	C_12_H_31_N_7_
143	19.703	318.30000	317.50362	C_18_H_39_NO_3_
144	19.73	362.32583	361.56617	C_20_H_43_NO_4_
145	19.879	256.25624	255.43859	
146	19.886	237.14136	236.34064	C_13_H_20_N_2_S
147	20.188	302.30455	301.49885	C_18_H_39_NO_2_
148	20.229	346.33073	345.57569	C_20_H_43_NO_3_
149	20.364	337.19662	336.42614	C_14_H_28_N_2_O_7_
150	20.938	386.32592	385.57069	C_22_H_43_NO_4_
151	20.947	330.33617	329.55028	C_20_H_43_NO_2_
152	21.010	374.36291	373.62298	C_22_H_47_NO_3_
153	21.085	301.13816	300.28852	C_13_H_20_N_2_
154	21.088	149.02325	148.11612	C_8_H_4_O_3_
155	21.340	388.34156	387.57253	C_22_H_45_NO_4_
156	21.389	234.96051	234.05388	C_9_H_2_N_2_O_2_S_2_
157	21.463	415.21075	414.49322	C_24_H_30_O_6_
158	21.466	586.29929	585.66389	C_28_H_39_N_7_O_7_
159	22.618	358.36684	357.62209	C_22_H_47_NO

**Table 2:** The extraction rate and purity of materials.

Material	Total Phenol Content/%	Phlorotannins Content/%	Purity/%
Brown algae dry powder	7.80	6.46	—
Phlorotannins extracts	34.07	25.16	73.85

## Data Availability

Not applicable.

## References

[1] Takeda H., Seki N. (1996). Enzyme-catalyzed Cross-linking and Degradation of Myosin Heavy Chain in Walleye Pollack Surimi Paste during Setting. Fish. Sci..

[2] Núñez-Flores R., Cando D., Borderías A.J., Moreno H.M. (2018). Importance of salt and temperature in myosin polymerization during surimi gelation. Food Chem..

[3] Hz A., Xiong Y., Bakry A.M., Xiong S., Yin T., Zhang B., Huang J., Liu Z., Huang Q. (2019). Effect of yeast β-glucan on gel properties, spatial structure and sensory characteristics of silver carp surimi. Food Hydrocoll..

[4] Zhang R., Zhang T., Hu M., Xue Y., Xue C. (2020). Effects of oleogels prepared with fish oil and beeswax on the gelation behaviors of protein recovered from Alaska Pollock. LWT.

[5] Hu Y., Shao Y., Wu C., Yuan C., Ishimura G., Liu W., Chen S. (2018). γ-PGA and MTGase improve the formation of ε-(γ-glutamyl) lysine cross-links within hairtail (*Trichiurus haumela*) surimi protein. Food Chem..

[6] Spanos G.A., Wrolstad R.E. (1992). Phenolics of apple, pear, and white grape juices and their changes with processing and storage. A review. J. Agric. Food Chem..

[7] Strauss G., Gibson S.M. (2004). Plant phenolics as cross-linkers of gelatin gels and gelatin-based coacervates for use as food in-gredients. Food Hydrocoll..

[8] Balange A.K., Benjakul S. (2009). Effect of oxidised phenolic compounds on the gel property of mackerel (*Rastrelliger kanagurta*) surimi. LWT-Food Sci. Technol..

[9] Buamard N., Benjakul S. (2019). Effect of ethanolic coconut husk extract and pre-emulsification on properties and stability of surimi gel fortified with seabass oil during refrigerated storage. LWT.

[10] Cho S., Shimizu M. (2015). Natural Sleep Aids and Polyphenols as Treatments for Insomnia. Bioactive Nutraceuticals and Dietary Supplements in Neurological and Brain Disease.

[11] Senapati S.R., Singh C.B., Hassan M.A., Vignaesh D., MartinXavier K.A., Balange A.K. (2016). Effect of differentsolvents on total phenolics and antioxidant activity of extracts from Sargassum tenerrium (J. Agardh, 1848). J. Environ. Bio.-Sci..

[12] Jiang D., Shen P., Pu Y., Jin M., Yu C., Qi H. (2020). Enhancement of gel properties of *Scomberomorus niphonius* myofibrillar protein using phlorotannin extracts under UVA irradiation. J. Food Sci..

[13] Shitole S.S., Balange A.K., Gangan S.S. (2014). Use of Seaweed (*Sargassum tenerrimum*) extract as gel enhancer for lesser sardine (*Sardinella brachiosoma*) surimi. Int. Aquat. Res..

[14] Shitole S., Balange A. (2014). Enhancement of gel strength of surimi from Japanese threadfin bream (*Nemipterus japonicus* Bloch, 1791) using seaweed extract. Fish. Technol..

[15] Wang Y., Tian X., Li J., Tu L., Tan S., Wu W., Bao B. (2018). Extraction Optimization Using Response Surface Methodology and Structure Identification of Phlorotannins from *Sargassum horneri*. Sci. Technol. Food Ind..

[16] Ferreres F., Lopes G., Gil-Izquierdo A., Andrade P.B., Sousa C., Mouga T., Valentão P. (2012). Phlorotannin Extracts from Fucales Characterized by HPLC-DAD-ESI-MS*^n^*: Approaches to Hyaluronidase Inhibitory Capacity and Antioxidant Properties. Mar. Drugs.

[17] Julavittayanukul O., Benjakul S., Visessanguan W. (2006). Effect of phosphate compounds on gel-forming ability of surimi from bigeye snapper (*Priacanthus tayenus*). Food Hydrocoll..

[18] Oujifard A., Benjakul S., Ahmad M., Seyfabadi J. (2012). Effect of bambara groundnut protein isolate on autolysis and gel properties of surimi from threadfin bream (*Nemipterus bleekeri*). LWT.

[19] Zhang M., Xu J., Zhu Y., Zhang R., Cheng Y., Jin Y. (2017). Effects of sucrose and glucose on the dielectric properties of minced Antarctic krill. Sci. Technol. Food Ind..

[20] Kroll J., Rawel H.M., Rohn S. (2003). Reactions of Plant Phenolics with Food Proteins and Enzymes under Special Consideration of Covalent Bonds. Food Sci. Technol. Res..

[21] Park J.W. (2013). Surimi and Surimi Seafood.

[22] Czubinski J., Dwiecki K. (2016). A review of methods used for investigation of protein-phenolic compound interactions. Int. J. Food Sci. Technol..

[23] Haslam E. (1989). Plant Polyphenols: Vegetable Tannins Revisited.

[24] Balange A.K., Benjakul S. (2009). Effect of oxidised tannic acid on the gel properties of mackerel (*Rastrelliger kanagurta*) mince and surimi prepared by different washing processes. Food Hydrocoll..

[25] Ngo V.P., Morioka K., Itoh Y. (2010). Microstructure of white croaker surimi protein gels set at low temperature under the in-hibition of the polymerization and degradation of protein. J. Biol. Sci..

[26] Shen P., Gao Z., Xu M., Rao J., Chen B. (2020). Physicochemical and structural properties of proteins extracted from dehulled industrial hempseeds: Role of defatting process and precipitation pH. Food Hydrocoll..

[27] Balange A.K., Benjakul S. (2009). Use of kiam wood extract as gel enhancer for mackerel (*Rastrelliger kanagurta*) surimi. Int. J. Food Sci. Technol..

[28] Maqsood S., Benjakul S., Shahidi F. (2013). Emerging Role of Phenolic Compounds as Natural Food Additives in Fish and Fish Products. Crit. Rev. Food Sci. Nutr..

[29] Frazier R.A., Papadopoulou A., Mueller-Harvey I., Kissoon D., Green R.J. (2003). Probing Protein−Tannin Interactions by Isothermal Titration Microcalorimetry. J. Agric. Food Chem..

[30] Nunes A., Bohigas X., Tejada J. (2006). Dielectric study of milk for frequencies between 1 and 20GHz. J. Food Eng..

[31] Zhu X.H., Guo W.C. (2010). A Review of Affecting Factors and Their Mechanisms of the Radio Frequency-Microwave Dielectric Properties of Foods. Food Sci..

[32] Schwan H.P. (1988). Biological effects of non-ionizing radiations: Cellular properties and interactions. Ann. Biomed. Eng..

[33] Mabrook M., Petty M. (2003). A novel technique for the detection of added water to full fat milk using single frequency admittance measurements. Sens. Actuators B Chem..

[34] Lizhi H., Toyoda K., Ihara I. (2008). Dielectric properties of edible oils and fatty acids as a function of frequency, temperature, moisture and composition. J. Food Eng..

[35] Cao H., Fan D., Jiao X., Huang J., Zhao J., Yan B., Zhou W., Zhang W., Zhang H. (2018). Heating surimi products using microwave combined with steam methods: Study on energy saving and quality. Innov. Food Sci. Emerg. Technol..

[36] Ryynänen S. (1995). The electromagnetic properties of food materials: A review of the basic principles. J. Food Eng..

[37] Damez J.-L., Clerjon S. (2013). Quantifying and predicting meat and meat products quality attributes using electromagnetic waves: An overview. Meat Sci..

[38] Clerjon S., Daudin J.-D., Damez J.-L. (2003). Water activity and dielectric properties of gels in the frequency range 200 MHz–6 GHz. Food Chem..

[39] Tananuwong K., Reid D. (2004). DSC and NMR relaxation studies of starch–water interactions during gelatinization. Carbohydr. Polym..

[40] Han M., Wang P., Xu X., Zhou G. (2014). Low-field NMR study of heat-induced gelation of pork myofibrillar proteins and its relationship with microstructural characteristics. Food Res. Int..

[41] Guo J., Zhou Y., Yang K., Yin X., Ma J., Li Z., Sun W., Han M. (2019). Effect of low-frequency magnetic field on the gel properties of pork myofibrillar proteins. Food Chem..

[42] Ducel V., Pouliquen D., Richard J., Boury F. (2008). 1H NMR relaxation studies of protein–polysaccharide mixtures. Int. J. Biol. Macromol..

[43] Mu Y., Sun J., Obadi M., Chen Z., Xu B. (2020). Effects of saccharides on the rheological and gelling properties and water mobility of egg white protein. Food Hydrocoll..

[44] Hills B., Takacs S., Belton P. (1989). The effects of proteins on the proton N.M.R. transverse relaxation times of water. Mol. Phys..

[45] Kuntz I., Kauzmann W. (1974). Hydration of proteins and polypeptides. Advances in Protein Chemistry.

[46] Chinachoti P., White V.A., Lo L., Stengle T.R. (1991). Application of high-resolution carbon-13, oxygen-17, and sodium-23 nuclear magnetic resonance to study the influences of water, sucrose, and sodium chloride on starch gelatinization. Cereal Chem..

[47] Dolata W., Piotrowska E., Wajdzik J., Tritt-Goc J. (2004). The use of the MRI technique in the evaluation of water distribution in tumbled porcine muscle. Meat Sci..

[48] Gianferri R., D’Aiuto V., Curini R., Delfini M., Brosio E. (2007). Proton NMR transverse relaxation measurements to study water dynamic states and age-related changes in Mozzarella di Bufala Campana cheese. Food Chem..

[49] Kudre T., Benjakul S., Kishimura H. (2013). Effects of protein isolates from black bean and mungbean on proteolysis and gel properties of surimi from sardine (*Sardinella albella*). LWT-Food Sci. Technol..

